# Deceased Organ Donation Registration and Familial Consent among Chinese and South Asians in Ontario, Canada

**DOI:** 10.1371/journal.pone.0124321

**Published:** 2015-07-31

**Authors:** Alvin Ho-ting Li, Eric McArthur, Janet Maclean, Cynthia Isenor, Versha Prakash, S. Joseph Kim, Greg Knoll, Baiju Shah, Amit X. Garg

**Affiliations:** 1 Department of Epidemiology & Biostatistics, Western University, London, Ontario, Canada; 2 Institute for Clinical Evaluative Sciences, Toronto, Ontario, Canada; 3 Trillium Gift of Life Network, Toronto, Ontario, Canada; 4 Division of Nephrology, Department of Medicine, University of Toronto, Toronto, Ontario, Canada; 5 Division of Nephrology and Ottawa Hospital Research Institute, The Ottawa Hospital and University of Ottawa, Ottawa, Ontario, Canada; 6 Department of Medicine, University of Toronto, Toronto, Ontario, Canada; Department of Transplantation and Renal Medicine, AUSTRALIA

## Abstract

**Objective:**

For various reasons, people of Chinese (China, Hong Kong or Taiwan) and South Asian (Indian subcontinent) ancestry (the two largest ethnic minority groups in Ontario, Canada) may be less likely to register for deceased organ donation than the general public, and their families may be less likely to consent for deceased organ donation at the time of death.

**Methods:**

We conducted two population-based studies: (1) a cross-sectional study of deceased organ donor registration as of May 2013, and (2) a cohort study of the steps in proceeding with deceased organ donation for patients who died in hospital from October 2008 to December 2012.

**Results:**

A total of 49 938 of 559 714 Chinese individuals (8.9%) and 47 774 of 374 291 South Asians (12.8%) were registered for deceased organ donation, proportions lower than the general public (2 676 260 of 10 548 249 (25.4%). Among the 168 703 Ontarians who died in a hospital, the families of 33 of 81 Chinese (40.1%; 95% CI: 30.7%-51.6%) and 39 of 72 South Asian individuals (54.2%; 95% CI: 42.7-65.2%) consented for deceased organ donation, proportions lower than the general public (68.3%; 95% CI: 66.4%-70.0%).

**Conclusions:**

In Ontario, Canada Chinese and South Asian individuals are less likely to register and their families are less likely to consent to deceased organ donation compared to the remaining general public. There is an opportunity to build support for organ and tissue donation in these two large ethnic communities in Canada.

## Introduction

There is a worldwide shortage of organs available for transplant. In 2012, almost 4000 Canadians were on a waiting list for an organ transplant and 230 died while waiting.[1] An immediate solution to this problem is to increase deceased organ donation consent rates, which in part is affected by the number of individuals registering their commitment to deceased organ donation in the advent of their death.[2] In Ontario, when the decedent is eligible, approximately 60% of families consent for deceased organ donation, and 23% of the population is registered for deceased organ donation.[3] Other provinces such as British Columbia and Quebec have less than 10% registered for deceased organ donation.[2]

While U.S studies have demonstrated that attitudes towards organ donation and consent rates are lower in black, Hispanic, Asian and older potential donors [4–7], these data may not generalize well to Canada’s population. In Canada, people of Chinese (China, Hong Kong, or Taiwan) and South Asian (Indian subcontinent) ancestry represent the two largest visible ethnic minority groups.[8] Previous studies from British Colombia and Alberta have suggested that these two groups are less likely to become deceased organ donors.[9–11] However, limitations of these studies include measuring support rather than actual registration, potential biases associated with survey design, and measuring ethnic differences in realized rather than eligible deceased organ donors.

We conducted two studies to test the hypotheses that Chinese and South Asians individuals in the province of Ontario, Canada are less likely to register for organ donation than the remaining general public (a cross-sectional study) and their families are less likely to consent to deceased organ donation at the time of death (a cohort study).

## Subjects and Methods

### Study design and setting

Using the large healthcare databases of Ontario, Canada, we conducted two population-based studies on Ontario citizens with a valid provincial health card number: 1) a retrospective cross-sectional study to examine the proportion of deceased organ donor registration and 2) a retrospective cohort study to examine rates of familial consent to deceased organ donation among South Asian individuals, Chinese individuals and the remaining general public. We conducted both studies at the Institute for Clinical Evaluative Sciences (ICES) according to a pre-specified protocol that was approved by the research ethics boards at Sunnybrook Health Sciences Centre (Toronto, Canada). This study follows reporting recommendations in the STROBE statement for observational studies (S1 Table).

As of 2008, Ontario’s organ and tissue donor registry became affirmative only (i.e. recording only ‘yes’ responses).[2] Individuals 16 years of age and older can register online or can mail in a consent registration form. It is also provincially mandated that individuals are asked about organ and tissue donor registration with all health-card related transactions, driver’s license renewals and Ontario photo ID applications at Service Ontario centres. Those who choose to register can select the option to exclude certain organs or tissues from donation.

### Data sources

We obtained the information used in both studies from three linked databases using coded identifiers.

First, we used the Ontario Registered Persons Database to identify the individual’s demographics and information on deceased organ donor registration. We derived the individual’s socioeconomic status using neighborhood income quintiles, (a household size-adjusted measure of income).[12]

Second, we obtained data from Ontario’s organ procurement organization, Trillium Gift of Life Network on all potential donors referred for consideration for deceased organ donation. Not all patients who die in a hospital have the potential for organ donation, and in Ontario hospitals with ventilator capacity can potentially make a referral. A ventilated patient who meets any of the following criteria is referred to Trillium Gift of Life Network to be considered as a potential organ donor: 1) grave prognosis or Glasgow Coma Scale score of 3; 2) injured brain or non-recoverable injury or illness; 3) family initiated discussion of donation or withdrawal of life sustaining therapy and 4) planned discussion of therapy limited, de-escalation of treatment or withdrawal of life sustaining therapy. All referred patients are then determined for medical suitability. This data is captured on a real-time basis by a call center. We did not include patients who were referred for tissue-only donation.

Third, we obtained information on diagnoses and procedures during hospitalization to ascertain the patient’s cause of death from the Canadian Institute for Health Information Discharge Abstract Database (CIHI-DAD). We classified cause of death using International Classification of Disease codes. We used similar codes from a previous study on deceased organ donation.[13] With the exception of neighbourhood income quintile (which was missing in less than 1% for both cohorts), the databases were complete for all variables used in this study.

### Individuals and outcomes

In the cross-sectional study, we studied Ontarians (>16 years of age and alive) as of May 17, 2013 to examine the proportion registered for deceased organ donation. In the cohort study, we studied all patients who died from October 25^th^ 2008 to December 31^st^ 2012 and all cases referred for deceased organ donation to Trillium Gift of Life Network to examine the rate of familial consent for deceased organ donation.

The primary outcome was whether the individual was registered for deceased organ and tissue donation (cross-sectional study) and whether the family provided consent for deceased organ donation (cohort study). In the cross-sectional study, we also examined the proportion of registrants that excluded specific organs (kidney, heart, liver, lung small bowel or pancreas) or tissues (eyes, bone, skin). In the cohort study, we assessed the primary outcome within 7 days of the decedent’s family being approached for donation.

### Ethnicity

We used a validated surname algorithm to identify individuals with South Asian or Chinese ancestry.[14] This algorithm has been used in several prior studies and demonstrates high positive predictive values when compared with self-reported ethnicity in a national survey (89.3% for South Asian and 91.9% for Chinese).[15–17] Among South Asians, the final list includes only names unique to South Asians (Hindu, Sikh and Sri Lankan surnames). Names used by South Asian Muslims or Christians were excluded because they could not be differentiated from people from other ethnic backgrounds such as Arab or Persian.[14]. Individuals whose surnames were not classified as South Asian or Chinese were categorized as the remaining general public.

### Statistical analysis

When the outcome is common, odds ratios estimated from cross-sectional and cohort data will overestimate the prevalence and rate ratio, respectively. Therefore, we used modified-Poisson regression to estimate prevalence (cross-sectional study) and rate ratio (retrospective-cohort study) along with their 95% confidence intervals.[18] We also used multivariable modified-Poisson regression to identify variables associated with organ donor registration, organ or tissue exclusion (among registrants only) and familial consent to deceased organ donation. We assessed baseline differences and compared proportions in deceased donor registration using standardized differences (cross-sectional study).[19] This metric describes differences between group means relative to the pooled standard deviation and is considered meaningful if a difference of greater than 10% is present. Deceased organ donor registration rates can vary by community, so we stratified the results according to whether an individual lived in the largest metropolitan area of the province (the Greater Toronto Area) or the rest of the province. We also assessed baseline differences of decedents whose family was approached for organ donation using an analysis of variance (ANOVA; retrospective-cohort study). We used the Wilson-score method to calculate 95% confidence intervals for proportions. We conducted all analysis with SAS software, version 9.3 (SAS Institute Incorporated, Cary, North Carolina, USA). In all outcome analyses, we interpreted two-tailed p-values <0.05 as statistically significant.

## Results

### Cross-sectional Study on Deceased Organ and Tissue Donor Registration

#### Baseline characteristics.

We identified 559 714 Chinese individuals, 374 291 South Asian individuals and 10 548 249 remaining general public who were eligible to register for deceased organ donation (S1 Fig). The characteristics of each group are listed in Table 1. Compared to the general public, Chinese and South Asian individuals were more likely to be from an urban city and of slightly lower socioeconomic status.

**Table 1:** Baseline Characteristics of Eligible Registrants (Cross-sectional Study).

Characteristic	Chinese (n = 559 714)	South Asians (n = 374 291)	General public (n = 10 548 249)	Standardized Differences^1^	
				Chinese	South Asians
**Mean age in years, (Standard Deviation)**	42.2 (17.7)	40.5 (17.2)	44.0 (19.2)	10%	19%
**Age**					
16–29 years	157413 (28.1%)	116085 (31.0%)	3015548 (28.6%)	1%	5%
30–39 years	109123 (19.5%)	85222 (22.8%)	1676407 (15.9%)	9%	18%
40–49 years	114039 (20.4%)	63667 (17.0%)	1798314 (17.0%)	9%	0%
50–59 years	81263 (14.5%)	47989 (12.8%)	1671152 (15.8%)	4%	9%
60–69 years	50423 (9.0%)	36226 (9.7%)	1208621 (11.5%)	8%	6%
≥ 70 years	47453 (8.5%)	25102 (6.7%)	1178207 (11.2%)	9%	16%
**Women**	294580 (52.6%)	187563 (50.1%)	5402949 (51.2%)	3%	2%
**Rural Residence** ^**2**^	3625 (0.6%)	2261 (0.6%)	1314886 (12.5%)	50%	50%
**Income Quintile** ^**3**^					
Fifth (Highest)	93 488 (16.7%)	40735 (10.9%)	2169377 (20.6%)	10%	27%
Fourth	110 826 (19.8%)	60634 (16.2%)	2184138 (20.7%)	2%	12%
Third (Middle)	109 947 (19.6%)	88606 (23.7%)	2075976 (19.7%)	0%	10%
Second	132 330 (23.6%)	92741 (24.8%)	2045928 (19.4%)	10%	13%
First (Lowest)	110 181 (19.7%)	91015 (24.3%)	2031718 (19.3%)	1%	12%
Missing	2942 (0.5%)	560 (0.1%)	41112 (0.4%)	1%	6%

^1^ Standardized Differences compared against the general public. Standardized Differences greater than 10% represent a meaningful difference between the two groups.

^2^ Refers to areas with population less than 10 000.

^3^ Categorized into fifths of average neighborhood income.

#### Organ donor registration

A total of 49 938 of 559 714 Chinese individuals (8.9%, 95% CI: 8.8%-9.0%) and 47 774 of 374 291 (12.8%, 95% CI: 12.7%-12.9%) South Asians were registered for deceased organ donation. These proportions were lower than the general public (2 676 260 of 10 548 249 were registered, [25.4%, 95% CI: 25.4%-25.4%]) (Table 2). Chinese were almost three times (Prevalence Ratio [PR], 0.35; 95% CI, 0.35–0.35) and South Asian individuals were two times (PR, 0.50; 95% CI, 0.50–0.51) less likely to register for deceased organ donation compared to the general public. These results were virtually unchanged after adjusting for age, sex, socioeconomic status and residency (urban vs. rural). Other factors associated with a higher likelihood of registering for deceased organ donation included women (vs. men), younger age (vs. older age), higher income (vs. lower income) and living in a rural (versus urban) location (Table 2).

**Table 2:** Factors associated with Donor Registration (Cross-sectional study).

Characteristic	No. Registered (%)	Adjusted Prevalence Ratio^1^ (95% CI)
**Ethnicity**		
Chinese	49 938 (8.9%)	0.35 (0.35 to 0.35)
South Asian	47 774 (12.8%)	0.50 (0.50 to 0.51)
General public	2 676 260 (25.4%)	1.00 [Reference]
**Residence**		
Urban	2 382 847 (23.4%)	1.00 [Reference]
Rural^2^	391 125 (29.6%)	1.25(1.25 to 1.26)
**Age Category**		
16–29 years	948 293 (28.8%)	1.00 [Reference]
30–39 years	558 946 (29.9%)	1.05(1.05 to 1.06)
40–49 years	496 331 (25.1%)	0.87(0.87 to 0.88)
50–59 years	394 863 (21.9%)	0.74(0.74 to 0.75)
60–69 years	246 021 (19%)	0.64(0.64 to 0.64)
≥ 70 years	129 518 (10.4%)	0.35(0.34 to 0.35)
**Sex**		
Men	1 250 333 (22.3%)	1.00 [Reference]
Women	1 523 639 (25.9%)	1.18(1.18 to 1.19)
**Income Quintile** ^**3**^		
Fifth (Highest)	640 973 (27.8%)	1.16 (1.16 to 1.17)
Fourth	585 153 (25.1%)	1.04 (1.03 to 1.04)
Three (Middle)	553 545 (23.9%)	1.00 [Reference]
Two	522 224 (23.0%)	0.98 (0.97 to 0.98)
One (Lowest)	472 077 (21.1%)	0.88 (0.88 to 0.88)

^1^Adjusted for Sex, Residency, Age, Income Quintile. Unadjusted prevalence ratios were essentially unchanged.

^2^ Refers to areas with population less than 10 000.

^3^ Categorized into fifths of average neighborhood income.

The results were similar when stratified either for those living in the largest metropolitan area (Greater Toronto Area) or the rest of the province (S2 Table). However, the absolute differences for deceased organ donor registration between the three groups was smaller in the Greater Toronto Area than the rest of the province (Greater Toronto Area: 8.0% [95% CI: 7.9%-8.1%] for Chinese and 11.8% [95% CI: 11.7%-12.0%] South Asian individuals were registered compared to 16.0% [95% CI: 15.9%-16.0%] for the remaining general public; Rest of the province: 12.9% [95% CI: 12.7%-13.1%] for Chinese and 17.0% [95% CI: 16.7%-17.3%] South Asian individuals compared to 30.1% [95% CI: 30.0%-30.1%] for the remaining general public).

When given the option to exclude certain organs and tissue (amongst those who had registered for organ donation), 9264 of 49 938 Chinese registrants (18.6%, 95% CI: 18.2%-18.9%), 11 889 of 47 774 South Asian registrants (24.9%, 95% CI: 24.5%-25.3%) and 412 487 of 2 676 260 general public registrants (15.4%, 95% CI: 15.4%-15.5%) excluded at least one organ or tissue (S3 Table). When adjusted as above, Chinese individuals (PR, 1.11; 95% CI, 1.09–1.13) and South Asian individuals (PR, 1.52; 95% CI, 1.50–1.55) were more likely to exclude an organ and/or tissue compared to the general public. Other factors associated with excluding an organ and/or tissue included men (vs. women), older (vs. younger) age, higher (vs. lower) socioeconomic status and living in a rural (vs. urban) city. Across the three groups, eyes and skin were the commonly excluded tissues. A relatively high proportion of South Asian individuals opted to exclude skin (17.5%). (S4 Table)

### Cohort Study on Familial Consent to Deceased Organ and Tissue Donation

#### Baseline characteristics

From October 25 2008 to December 31 2012, a total of 168 703 Ontarians died in a hospital (S2 Fig). A total of 5581 of these Ontarians were referred for deceased organ and tissue donation. Of those referred, the families of 81 Chinese decedents, 72 South Asian decedents and 2558 remaining general public decedents were approached to obtain familial consent for organ donation. The baseline characteristics of the decedents approached for donation are listed in Table 3. Compared to the general public, Chinese and South Asian decedents had significantly different causes of deaths.

**Table 3:** Baseline characteristics of decedents whose family was approached for organ donation^1^ (Cohort study).

Characteristic	Chinese (n = 81)	South Asian (n = 72)	General public (n = 2558)	*P*
**Mean age in years, (Standard Deviation)**	57.9 (19.27)	48.7 (18.72)	53.7 (18.75)	<0.05
**Age**				
0–44	15 (18.5%)	25 (34.7%)	662 (25.9%)	<0.05
45–54	13 (16.0%)	17 (23.6%)	481 (18.8%)	
55–64	21 (25.9%)	19 (26.4%)	587 (22.9%)	
65+	32 (39.5%)	11 (15.3%)	828 (32.4%)	
**Women**	29 (35.8%)	29 (40.3%)	1047 (40.9%)	0.65
**Rural Residency** ^**2**^	< = 5 (2.5%)	< = 5 (6.9%)	334 (13.1%)	<0.01
**Income Quintile** ^**3**^				0.23
Quintile 5 (highest)	17 (21.0%)	17 (23.6%)	571 (22.3%)	
Quintile 4	17 (21.0%)	24 (33.3%)	547 (21.4%)	
Quintile 3	19 (23.5%)	16 (22.2%)	516 (20.2%)	
Quintile 2	17 (21.0%)	9 (12.5%)	502 (19.6%)	
Quintile 1 (lowest)	11 (13.6%)	6 (8.3%)	422 (16.5%)	
**Cause of Death**				<0.01
Traumatic Brain Injury	16 (19.8%)	17 (23.6%)	435 (17.0%)	
Subarachnoid/Intracerebral Hemorrhagic event	35 (43.2%)	16 (22.2%)	620 (24.2%)	
Other damage to the brain^4^	17 (21.0%)	21 (29.2%)	643 (25.1%)	
All other causes of death^5^	13 (16.0%)	18 (25.0%)	860 (33.6%)	

^1^ Cell sizes less than or equal to 5 are suppressed to protect confidentiality. Several categories were collapsed to protect confidentiality.

^2^ Refers to areas with population less than 10 000.

^3^ Categorized into fifths of average neighborhood income.

^4^ Includes anoxic brain damage, cerebral edema, cerebral infarction, cerebral thrombosis and asphyxiation, and other disorders of the brain.

^5^ Includes cardiac arrest and acute myocardial infarction.

#### Consent for organ donation

Overall, 68.3% (95% Confidence interval [CI]: 66.4%-70.0%) of general public families consented for deceased organ donation when approached compared to 40.7% (95% CI: 30.7%-51.6%) of Chinese families and 54.2% (95% CI: 42.7%-65.2%) of South Asian families (Table 4). Families of Chinese decedents (Rate Ratio [RR], 0.60; 95% CI: 0.46–0.78) or families of South Asian decedents (RR, 0.79; 95% CI: 0.64–0.98) were less likely to provide consent for deceased organ donation compared to the general public. Results were not appreciably different after adjusting for sex, residency (urban vs. rural), age, socioeconomic status and cause of death (Table 4). When looking at the other factors associated with a higher likelihood of consent for deceased organ donation, families of older decedents (55+ years old) were less likely to consent compared to younger decedents (18–34 years old) (Table 4). Families of decedents with other causes of death were less likely to provide consent compared to those who died from traumatic brain injury (RR, 0.86; 95% CI: 0.80–0.92).

**Table 4:** Factors associated with Familial Consent (Cohort Study).

Characteristic	Number of Individuals consented (%)	Rate Ratio (95% CI)	
		Unadjusted	Adjusted^1^
**Ethnicity**			
General public	1746 (68.3%)	1.00 [Reference]	1.00 [Reference]
Chinese	33 (40.7%)	0.60 (0.46 to 0.78)	0.62 (0.48 to 0.80)
South Asian	39 (54.2%)	0.79 (0.64 to 0.98)	0.77 (0.63 to 0.96)
**Sex**			
Men	1083 (67.4%)	1.00 [Reference]	1.00 [Reference]
Women	735 (66.5%)	0.99 (0.93 to 1.04)	0.99 (0.94 to 1.04)
**Residency** ^**2**^			
Urban	1578 (66.4%)	1.00 [Reference]	1.00 [Reference]
Rural	98 (29.0%)	1.07 (0.99 to 1.15)	1.03 (0.96 to 1.10)
**Age**			
0–18	103 (72.5%)	0.94 (0.83 to 1.05)	0.96 (0.86 to 1.08)
18–34	228 (77.6%)	1.00 [Reference]	1.00 [Reference]
35–44	198 (74.4%)	0.96 (0.87 to 1.05)	0.97 (0.89 to 1.07)
45–54	374 (73.2%)	0.94 (0.87 to 1.02)	0.96 (0.88 to 1.04)
55–64	427 (68.1%)	0.88 (0.81 to 0.95)	0.89 (0.82 to 0.97)
65–74	346 (60.8%)	0.78 (0.72 to 0.86)	0.80 (0.73 to 0.88)
75+	142 (47.0%)	0.61 (0.53 to 0.69)	0.62 (0.54 to 0.71)
**Income Quintile** ^**3**^			
Quintile 5 (highest)	317 (72.2%)	1.08 (1.00 to 1.17)	1.08 (1.00 to 1.18)
Quintile 4	371 (70.3%)	1.05 (0.97 to 1.14)	1.05 (0.97 to 1.13)
Quintile 3	368 (66.8%)	1.00 [Reference]	1.00 [Reference]
Quintile 2	388 (66.0%)	0.99 (0.91 to 1.07)	0.99 (0.91 to 1.07)
Quintile 1 (lowest)	374 (61.8%)	0.93 (0.85 to 1.01)	0.93 (0.85 to 1.01)
**Cause of Death**			
Traumatic Brain Injury	348 (74.4%)	1.00 [Reference]	1.00 [Reference]
Subarachnoid Hemorrahage events	207 (77.5%)	1.04 (0.96 to 1.13)	1.05 (0.96 to 1.14)
Intracerebral Hemorrhage	253 (62.6%)	0.84 (0.77 to 0.92)	0.91 (0.82 to 1.00)
Other damage to the brain^4^	292 (68.2%)	0.92 (0.84 to 1.00)	0.92 (0.85 to 1.00)
Acute Myocardial Infarction, Cardiac Arrest	164 (64.8%)	0.87 (0.78 to 0.97)	0.90 (0.81 to 1.00)
All other causes of death^5^	554 (62.2%)	0.84 (0.78 to 0.90)	0.86 (0.80 to 0.92)

**Note:** CI = confidence interval.

^1^Adjusted for Sex, Residency, Age, Income Quintile, Cause of Death.

^2^ Refers to areas with population less than 10 000.

^3^ Categorized into fifths of average neighborhood income.

^4^ Includes anoxic brain damage, cerebral edema, cerebral infarction, cerebral thrombosis and asphyxiation, other disorders of the brain.

^5^ Includes cardiac arrest and acute myocardial infarction.

## Discussion

We found that Chinese and South Asian Ontarians had lower deceased organ donor registration and consent rates compared to the remaining general public.

Our findings are consistent with other studies demonstrating that Chinese and South Asians are less likely to be organ and tissue donors. Although we found large differences in donor registrations between the three groups, differences in familial consent were smaller. The low organ donor registration may be in part due to the lack of awareness of the provincial donor registry.[20,21] Among ethnic minorities in North America and the United Kingdom, a recent review found that there was less favourable cultural/religious beliefs towards organ/tissue donation as well as less trust in healthcare professionals and the organ allocation system.[22] Further research on culturally-sensitive strategies to raise awareness and promote organ donation is warranted. For example, we found that many South Asian registrants opted to exclude skin for donation, which may have been affected by the myth that organ donation will disfigure the donor’s body.[23] Finally, a US study revealed that most organ procurement organizations (90%) estimate that less than 10% of families of registered organ donors objected to deceased organ donation.[24] Therefore, increasing the number of registrants may be an important strategy to build support for organ and tissue donation and increase consent rates. Such a strategy has proven successful in other contexts. For example, in the United States, multiple educational campaigns including media campaigns, and educational programs at high schools and churches significantly improved the Hispanic American population’s awareness, knowledge and intention to donate organs.[25] In addition, an aggressive outreach program implemented at high schools, churches, and medical clinics increased consent rate among Hispanic Americans from 56% in 2005 to 83% in 2011 (P = 0.004) [26]. According to a recent review, community-based educational programs are more effective at increasing registration for organ donation among ethnic minorities compared to mass media campaigns.[27] To be successful, the program should be delivered by local community members in familiar environments and include a strong interpersonal element that addresses specific concerns of the community.[27] Giving the community ownership of the health issue may also be more effective than alternative approaches.[28]

### Strengths and limitations

Our study examined deceased organ and tissue donor registration and familial consent among the two largest visible ethnic minorities in the entire province of Ontario, Canada. To our knowledge, our study is the first to document actual registration rates among ethnic minorities, rather than expressed support to donate. Further, we had data on all eligible deaths, referrals for deceased organ donation and whether the family was actually approached to obtain consent for deceased organ donation.

However, our study has some limitations. First, we did not identify any barriers to organ donation and had no information on the reasons why Chinese and South Asian individuals did not register for deceased donation or provide familial consent for organ donation, which would be useful to inform educational programs tailored for ethnicity. Second, we identified Chinese and South Asians based on a validated list of Chinese surnames with high positive predictive value but low sensitivity. There is the potential for misclassifying individuals whose surnames do not reflect their ethnicity. Third, this study focused only on Chinese and South Asian individuals. Future use of other data sources, including the immigration and First Nations databases, would provide opportunities to examine similar issues in other ethnic groups. Fourth, we were not able to distinguish whether patients were eligible for donation after brain death or donation after circulatory death. It may be possible that familial consent is influenced by cultural differences in the understanding of death. Finally, we estimate our general public group is made up of approximately 85% of individuals with European ancestry.[8] Aboriginal and Afro-Caribbean individuals share many surnames with the European population and are classified as the general public in this study.[22] Finally, we examined deceased organ donor registration for the Ontario population and stratified the results by the largest metropolitan area. Although ethnic communities have the same access to information about organ donation, registration rates can be influenced by level and type of organ donor registry awareness activities within each community. Further, other factors that could influence organ donor registration such as religious beliefs[29], education[30], medical mistrust[31,32], immigration status, and concerns about recording their identity in a government database were not measured in our study.

This study demonstrates that Chinese and South Asian Ontarians have lower deceased organ donor registration and familial consent rates compared to the general public. There is an opportunity to build support for organ and tissue donation in these large ethnic communities, which could help more patients receive a life-saving transplant and reduce their time on the waiting list.

## Supporting Information

S1 Fig Selection of participants for inclusion in the Cross-sectional study on Deceased Organ Donor Registration. (DOCX)

S2 Fig Selection of participants for inclusion in the Retrospective Cohort Study on Familial Consent. (DOCX)

S1 Table Checklist of recommendations for reporting of observational studies using the STROBE guidelines. (DOCX)

S2 Table Factors associated with donor registration stratified by the largest metropolitan area (Cross-sectional study). (DOCX)

S3 Table Factors associated with opting-out an organ and/or tissue among donor registrants (Cross-sectional study). (DOCX)

S4 Table Proportions of registered organ and tissue donors excluding organs and/or tissues. (DOCX)
