# Supplementary material for: Deceased Organ Donation Registration and Familial Consent among Chinese and South Asians in Ontario, Canada
Source: PLoS One. 2015 Jul 31;10(7):e0124321. doi: 10.1371/journal.pone.0124321 (PMC4521812; doi:10.1371/journal.pone.0124321)
Supplement: S1 Fig — (DOCX) [file pone.0124321.s001.docx]

**Figure S1:** Selection of participants for inclusion in the cross-sectional study on deceased organ donor registration

| ^1^Data cleaning steps included invalid patient identifier, missing sex, non-Ontarian, death date prior to index date  ^2^This exclusion was applied to ensure that the individual was living in Ontario |
| --- |

Everyone in the Ontario Registered Persons Database (**n=17 424 428)**

Exclusions **(n=5 942 174)**

Data cleaning steps^1^: 2 366 019

Less than 16 years of age: 2 225 793

Date of last contact > 5 years^2^: 1 350 362

Ontarians included in study **(n=11 482 254)**

Registered for deceased donation: 2 773 972 (24%)

Not registered: 8 708 282 (86%)

After applying last name algorithm:

Chinese: 559 714

South Asian: 374 291

General Public: 10 548 249
