# Supplementary material for: Deceased Organ Donation Registration and Familial Consent among Chinese and South Asians in Ontario, Canada
Source: PLoS One. 2015 Jul 31;10(7):e0124321. doi: 10.1371/journal.pone.0124321 (PMC4521812; doi:10.1371/journal.pone.0124321)
Supplement: S2 Fig — (DOCX) [file pone.0124321.s002.docx]

**Figure S2:** Selection of participants for inclusion in the Retrospective- cohort study on familial consent

| ^1^Data cleaning steps included invalid patient identifier, missing sex, non-Ontarian, death date prior to index date  ^2^This exclusion was applied to ensure that the individual was living in Ontario |
| --- |

After applying last name algorithm:

Chinese: 81

South Asian: 72

General Public: 2558

Number of Ontarians whose families were approached for organ donation:

Approached: 2711

Not approached: 3110

Ontarians referred for deceased organ donation (**n=5581**)

Patients who died between October 25 2008 to December 31 2012 (**n=364 610)**

Exclusions **(n=195 907)**

Data cleaning steps^1^: 525

Death outside of hospital: 195 382
