# Supplementary material for: Deceased Organ Donation Registration and Familial Consent among Chinese and South Asians in Ontario, Canada
Source: PLoS One. 2015 Jul 31;10(7):e0124321. doi: 10.1371/journal.pone.0124321 (PMC4521812; doi:10.1371/journal.pone.0124321)
Supplement: S2 Table — (DOCX) [file pone.0124321.s004.docx]

**Table S2:** Factors associated with Donor Registration (Cross-sectional study)

|  | **Largest Metropolitan Area (Greater Toronto Area)**  (n=4 271 087) | | **Rest of the Province**  (n=7 211 167) | |
| --- | --- | --- | --- | --- |
| **Characteristic** | **No. Registered (%)** | **Adjusted Prevalence Ratio^1^**  (95% CI) | **No. Registered (%)** | **Adjusted Prevalence Ratio^2^**  (95% CI) |
| **Ethnicity** |  |  |  |  |
| Chinese | 36 375 (8.0%) | 0.50 (0.49 to 0.50) | 13 563 (12.9%) | 0.41 (0.40 to 0.41) |
| South Asian | 36 352 (11.8%) | 0.75 (0.74 to 0.76) | 11 422 (17.0%) | 0.53 (0.52 to 0.54) |
| General public | 559 927 (16.0%) | 1.00 [Reference] | 2 116 333 (30.1%) | 1.00 [Reference] |
| **Residence** |  |  |  |  |
| Urban | Not applicable | Not applicable | 1 750 193 (29.7%) | 1.00 [Reference] |
| Rural^3^ | Not applicable | Not applicable | 391 125 (29.6%) | 1.02 (1.02 to 1.02) |
| **Age Category** |  |  |  |  |
| 16 – 29 years | 224 334 (18.5%) | 1.00 [Reference] | 723 959 (34.9%) | 1.00 [Reference] |
| 30 – 39 years | 156 555 (19.7%) | 1.08 (1.07 to 1.08) | 402 391 (19.4%) | 1.08 (1.08 to 1.08) |
| 40 – 49 years | 110 346 (14.5%) | 0.79 (0.78 to 0.79) | 385 985 (35.9%) | 0.91 (0.91 to 0.92) |
| 50 – 59 years | 76 108 (12.0%) | 0.63 (0.63 to 0.64) | 318 755 (26.2%) | 0.78 (0.78 to 0.78) |
| 60 – 69 years | 42 374 (9.6%) | 0.50 (0.50 to 0.50) | 203 647 (17.5%) | 0.68 (0.67 to 0.68) |
| ≥ 70 years | 22 937 (5.4%) | 0.28 (0.27 to 0.28) | 106 581 (12.9%) | 0.36 (0.36 to 0.37) |
| **Sex** |  |  |  |  |
| Men | 283 196 (13.8%) | 1.00 [Reference] | 967 137 (27.3%) | 1.00 [Reference] |
| Women | 349 458 (15.8%) | 1.17 (1.16 to 1.17) | 1 174 181 (32.0%) | 1.19 (1.19 to 1.20) |
| **Income Quintile^4^** |  |  |  |  |
| Fifth (Highest) | 148 418 (20.3%) | 1.39 (1.38 to 1.40) | 492 555 (31.3%) | 1.07 (1.07 to 1.07) |
| Fourth | 127 352 (16.1%) | 1.09 (1.09 to 1.10) | 457 801 (29.3%) | 0.99 (0.99 to 0.99) |
| Three (Middle) | 131 547 (14.8%) | 1.00 [Reference] | 421 998 (29.5%) | 1.00 [Reference] |
| Two | 121 312 (13.1%) | 0.90 (0.89 to 0.90) | 400 912 (29.9%) | 1.01 (1.01 to 1.02) |
| One (Lowest) | 104 025 (11.2%) | 0.74 (0.74 to 0.75) | 368 052 (28.2%) | 0.95 (0.95 to 0.95) |
| ^1^Adjusted for Sex, Residency, Age, Income Quintile. Unadjusted prevalence ratios were essentially unchanged.  ^2^ Adjusted for Sex, Age, Income Quintile. Unadjusted prevalence ratios were essentially unchanged.  ^3^ Refers to areas with population less than 10 000.  ^4^ Categorized into fifths of average neighborhood income | | | | |
