# Supplementary material for: Deceased Organ Donation Registration and Familial Consent among Chinese and South Asians in Ontario, Canada
Source: PLoS One. 2015 Jul 31;10(7):e0124321. doi: 10.1371/journal.pone.0124321 (PMC4521812; doi:10.1371/journal.pone.0124321)
Supplement: S3 Table — (DOCX) [file pone.0124321.s005.docx]

**Table S3.** Factors associated with opting-out of at least one organ and/or tissue among donor registrants (Cross-sectional study)

| **Characteristic** | **No. Registered Opting-out (%)** | **Prevalence Ratio (95% CI)** | |
| --- | --- | --- | --- |
|  |  | **Unadjusted** | **Adjusted^1^** |
| **Ethnicity** |  |  |  |
| Chinese | 9264 (18.6%) | 1.20 (1.18 to 1.23) | 1.11 (1.09 to 1.13) |
| South Asian | 11889 (24.9%) | 1.61 (1.59 to 1.64) | 1.52 (1.50 to 1.55) |
| General public | 412487 (15.4%) | 1.00 [Reference] | 1.00 [Reference] |
| **Residence** |  |  |  |
| Urban | 380 250 (16%) | 1.00 [Reference] | 1.00 [Reference] |
| Rural^2^ | 53 390 (13.7%) | 0.86 (0.85 to 0.86) | 0.92 (0.91 to 0.93) |
| **Age Category** |  |  |  |
| 16 – 29 years | 208 567 (22%) | 1.00 [Reference] | 1.00 [Reference] |
| 30 – 39 years | 90 725 (16.2%) | 0.74 (0.73 to 0.74) | 0.74 (0.74 to 0.75) |
| 40 – 49 years | 62226 (12.5%) | 0.57 (0.57 to 0.57) | 0.58 (0.58 to 0.59) |
| 50 – 59 years | 40534 (10.3%) | 0.47 (0.46 to 0.47) | 0.48 (0.48 to 0.49) |
| 60 – 69 years | 21595 (8.8%) | 0.4 (0.39 to 0.4) | 0.42 (0.41 to 0.42) |
| ≥ 70 years | 9993 (7.7%) | 0.35 (0.34 to 0.36) | 0.37 (0.36 to 0.37) |
| **Sex** |  |  |  |
| Men | 164855 (13.2%) | 1.00 [Reference] | 1.00 [Reference] |
| Women | 268785 (17.6%) | 1.34 (1.33 to 1.35) | 1.27 (1.26 to 1.27) |
| **Income Quintile^3^** |  |  |  |
| Fifth (Highest) | 94181 (14.7%) | 0.93 (0.92 to 0.94) | 0.96 (0.95 to 0.97) |
| Fourth | 90747 (15.5%) | 0.98 (0.98 to 0.99) | 1.00 (0.99 to 1.00) |
| Three (Middle) | 87190 (15.8%) | 1.00 [Reference] | 1.00 [Reference] |
| Two | 83181 (15.9%) | 1.01 (1.00 to 1.02) | 1.00 (0.99 to 1.01) |
| One (Lowest) | 78341 (16.6%) | 1.05 (1.04 to 1.06) | 1.03 (1.02 to 1.04) |
| **Note:** CI= confidence interval  ^1^Adjusted for Sex, Residency, Age, Income Quintile.  ^2^ Refers to areas with population less than 10 000.  ^3^ Categorized into fifths of average neighborhood income. | | | |
