# Supplementary material for: Deceased Organ Donation Registration and Familial Consent among Chinese and South Asians in Ontario, Canada
Source: PLoS One. 2015 Jul 31;10(7):e0124321. doi: 10.1371/journal.pone.0124321 (PMC4521812; doi:10.1371/journal.pone.0124321)
Supplement: S4 Table — (DOCX) [file pone.0124321.s006.docx]

**Table S4.** Proportions of registered organ and tissue donors excluding organs and/or tissues

| **Organ and/or Tissue:** | **Number of Registrants that opted-out:** | | |
| --- | --- | --- | --- |
|  | **Chinese**  (n=27 563) | **South Asian**  (n=23 535) | **General Public**  (n=1 472 541) |
| Kidney | 947 (1.9%) | 2381 (5.0%) | 26 334 (1.0%) |
| Heart | 1724 (3.5%) | 2857 (6.0%) | 43 496 (1.6%) |
| Eyes | 5571 (11.2%) | 5486 (11.5%) | 270 430 (10.1%) |
| Bone | 3754 (7.5%) | 6121 (12.8%) | 131 359 (4.9%) |
| Liver | 1042 (2.1%) | 2714 (5.7%) | 28 399 (1.1%) |
| Lung | 1102 (2.2%) | 3037 (6.4%) | 43 678 (1.6%) |
| Skin | 5573 (11.2%) | 8344 (17.5%) | 242 573 (9.1%) |
| Pancreas | 1529 (3.1%) | 3887 (8.1%) | 49 157 (1.8%) |
| Any of the Above | 9264 (18.6%) | 11889 (24.9%) | 412 487 (15.4%) |
